# Detailed investigation of the composition and transformations of phenolic compounds in fresh and fermented *Vaccinium floribundum* berry extracts by high‐resolution mass spectrometry and bioinformatics

**DOI:** 10.1002/pca.3105

**Published:** 2022-01-21

**Authors:** Andrea Cerrato, Susy Piovesana, Sara Elsa Aita, Chiara Cavaliere, Simona Felletti, Aldo Laganà, Carmela Maria Montone, Celia Vargas‐de‐la‐Cruz, Anna Laura Capriotti

**Affiliations:** ^1^ Department of Chemistry Sapienza University of Rome Rome Italy; ^2^ Department of Chemistry and Pharmaceutical Sciences University of Ferrara Ferrara Italy; ^3^ CNR NANOTEC, Campus Ecotekne University of Salento Lecce Italy; ^4^ Faculty of Pharmacy and Biochemistry, Academic Department Pharmacology, Toxicology and Bromatology Centro Latinoamericano de Enseñanza e Investigación en Bacteriología Alimentaria‐CLEIBA, Universidad Nacional Mayor de San Marcos Lima Peru

**Keywords:** blueberry, Compound Discoverer, high‐resolution mass spectrometry, Neotropical berries, polyphenols, quercetin quinone

## Abstract

**Introduction:**

Blueberries are known for their very high content of biologically active phenolic compounds; nonetheless, differently from the North American and European species of blueberries, Neotropical blueberries have not been extensively studied yet.

**Objectives:**

In the present paper, the phenolic composition of *Vaccinium floribundum* Kunth, which is endemic to the Andean regions and grows 1,600 to 4,500 meters above sea level, was investigated by ultra‐high‐performance liquid chromatography coupled to high‐resolution mass spectrometry (UHPLC‐HRMS). Native and fermented berries were compared in terms of phenolic composition as well as antioxidant activity, total phenolic content, and total anthocyanin content.

**Materials and Methods:**

*V. floribundum* native and fermented berries were extracted and analyzed by UHPLC‐HRMS. The acquired datasets were processed by Compound Discoverer 3.1 using a dedicated data analysis workflow that was specifically set up for phenolic compound identification.

**Results:**

In total, 309 compounds were tentatively identified, including anthocyanins, flavonoids, phenolic acids, and proanthocyanidins. The molecular transformations of phenolic compounds during fermentation were comprehensively investigated for the first time, and by a customized data processing workflow, 13 quinones and quinone methides were tentatively identified in the fermented samples. Compared to other species of the genus *Vaccinium*, a peculiar phenolic profile is observed, with low abundance of highly methylated compounds.

**Conclusion:**

Andean berries are a rich source of a wide variety of phenolic compounds. Untargeted MS analyses coupled to a dedicated data processing workflow allowed expanding the current knowledge on these berries, improving our understanding of the fate of phenolic compounds after fermentation.

## Abstract 2

In the present paper, the phenolic composition of *Vaccinium floribundum* Kunth (endemic to the Andean regions) was investigated by ultra‐high‐performance liquid chromatography coupled to high‐resolution mass spectrometry; 309 compounds were tentatively identified. After lactic fermentation, fresh and fermented berries were compared in terms of antioxidant activity, total phenolic content, and total anthocyanin content. Moreover, the molecular transformations of phenolic compounds during fermentation were comprehensively investigated for the first time, providing the tentative identification of 13 quinones and quinone methides.

## 1 INTRODUCTION

Increased intake of fruits like berries, rich in nutrients and phytochemicals, is recommended in dietary guidelines for their beneficial health effects.^1^ Consumption of berry fruits is not only limited to fresh or frozen forms, as several processed and derived products are prepared, such as dried and fermented berries, yogurts, beverages, and jams.^2^ Moreover, in recent years, berry extracts have been increasingly employed as functional food and dietary supplements combined with other vegetable and herbal extracts.^3^ Raspberry extracts have been demonstrated to exert antineuroinflammatory effects,^4^ while anthocyanin‐rich strawberry extracts were shown to protect human dermal fibroblasts against oxidative damage.^5^ Berries belonging to the genus *Vaccinium*, such as European blueberry (also known as bilberry or huckleberry, *Vaccinium myrtillus*) and North American blueberry (*Vaccinium corymbosum*), have received increasing interest due to their extremely high content of flavonoids, anthocyanins, phenolic acids, and tannins, which have been demonstrated to exert a wide range of biological activities.^6^ In a recent paper by Rutledge *et al*.,^7^ blueberry phenolics were associated with cognitive enhancement in healthy older adults. Likewise, Stull^8^ reported that consumption of whole blueberries reduces the blood glucose level in vivo. For these reasons, blueberry is often referred to as a "superfruit." The composition of phenolic compounds in North American and European blueberries has been widely studied.^9^, ^10^ Ancillotti *et al*.^11^ reported more than 200 compounds in blueberry hydroalcoholic extracts, comprising mainly anthocyanins, flavonols, and proanthocyanidins (PACs), and described the differences among *V. myrtillus*, *V. corymbosum*, and *Vaccinium uliginosum*. Other than *V. myrtillus*, there are several other lesser‐known wild species of the genus *Vaccinium*, such as *Vaccinium floribundum* Kunth, known with the trivial names of Andean blueberry or mortiño. *V. floribundum* is a woody perennial shrub that is endemic to the Andean region in South America, ranging from Venezuela to Bolivia, and can be found between 1,600 to 4,500 meters above sea level.^12^ Mortiño is known to play a significant environmental and ecological role, being one of the first species that recover after bouts of deforestation and human‐made fires.^12^ Moreover, fruits have high concentrations of phenolic compounds with potential beneficial effects on human health and are widely used by the local population in native and fermented form or for the preparation of traditional drinks, ice creams, preserves, and wines.^13^ Despite the growing interest in the bioactive compounds in berries from South America,^14^ there is still a lack of knowledge on the phenolic compound composition of *V. floribundum*. The few papers dealing with the identification of phenolic compounds are limited to specific subclasses and use low‐resolution techniques.^15^, ^16^, ^17^, ^18^ In the present article, *V. floribundum* Kunth from the Peruvian Andean Region was characterized by high‐resolution mass spectrometry (HRMS), which is the foremost technique for untargeted analysis of phenolic compounds.^19^ Moreover, as wild edible berries are often fermented before consumption to enhance phenolic compounds' bioavailability,^20^ *V. floribundum* berries were subjected to lactic fermentation by *Lactobacillus plantarum* to compare its biological activities to those of the native berry. Moreover, the transformations of the phenolic molecular species were comprehensively evaluated for the first time. For this purpose, a semi‐automated data processing workflow for the identification of phenolic degradation products was set up.

## 2 EXPERIMENTAL

### 2.1 Chemicals and bacterial strains

*V. floribundum* Kunth fruit samples were collected between March and April 2019 in Sanchez‐Carriòn province (La Libertad, Peru) and gathered at the National University of San Marcos (Lima, Peru), where their taxonomy was certified by the Herbario San Marcos (National University of San Marcos, Lima, Peru). Berries were mashed, freeze‐dried by a Heto PowerDry LL1500 (Thermo Fisher), finely ground in a mortar, and stored at −20°C until use. Optima® MS grade water, methanol (MeOH), and acetonitrile (ACN) were purchased from Thermo Fisher Scientific (Waltham, Massachusetts, USA). Acetone, acetic acid, formic acid, sodium hypochlorite, sodium acetate, 2,2′‐azino‐bis(3‐ethylbenzothiazoline‐6‐sulfonic acid) diammonium salt (ABTS), and Folin–Ciocalteu's phenol reagent were purchased from Merck (Kenilworth, New Jersey, USA). *L. plantarum* starters were supplied by the American Type Culture Collection (ATCC, Manassas, Virginia, USA). De Man, Rogosa, and Sharpe (MRS) agar powder and yeast from *Saccharomyces cerevisiae* were purchased from Sigma‐Aldrich (St. Louis, Missouri, USA). *L. plantarum* at 5% was activated in 10 mL of MRS agar broth for 16 h at 30°C in anaerobic conditions, diluted to 1% with an additional 40 mL MRS agar broth, and activated for a further 16 h under the same conditions.

### 2.2 Fermentation process

In total, 166 g of frozen *V. floribundum* Kunth berries was disinfected with a diluted sodium hypochlorite solution and washed three times with distilled water. The berries were added to 500 mL of ultra‐pure water, liquefied, and placed in an amber bottle. Sterilization of the berries was performed by the thermal shock method: the homogenized material was subjected to a temperature of 70°C for 10 min, placed in an ice bath until it reached a temperature of 40°C, subjected to a temperature of 85°C for 5 min, and finally placed in an ice bath until it reached room temperature. Next, 4% yeast was added to the homogenized material to a final concentration of 0.4%, and then *L. plantarum* inoculum was added to a final concentration of 0.01%. Fermentation was carried out at 30°C, for 48 h, in the dark and under constant stirring. Aliquots of 20 mL of the product were freeze‐dried by a Heto PowerDry LL1500 (Thermo Fisher).

### 2.3 Phenolic compound extraction

Fresh and fermented berries were extracted as previously reported with slight modifications.^21^ Briefly, 0.2 g of freeze‐dried berry samples was extracted with 10 mL CH_3_COCH_3_/H_2_O/CH_3_COOH (70:29.5:0.5, v/v/v). The extract was sonicated for 15 min in an ice bath and then centrifuged for 10 min at 2000 ×*g* using an Elmasonic S 60 H (Elma, Singen, Germany). The supernatant was collected, and the procedure was repeated once. The supernatants were mixed (20 mL) and concentrated to 4.5 mL using a Speed‐Vac SC 250 Express (Thermo 164 Avant, Holbrook, NY, USA). Then, 500 μL of MeOH was added to the sample, and the final extract solution (H_2_O/MeOH, 90:10, v/v) was filtered through a 13‐mm Acrodisc Syringe filter with a 0.2 μm GH Polypro membrane (Pall, Ann Arbor, MI, USA). Finally, the extract was aliquoted and stored at −20°C until further analysis. For each sample, three biological replicates were carried out.

### 2.4 Determination of the antioxidant activity

The ABTS antioxidant assay was carried out as previously described with slight modifications.^22^ ABTS was dissolved in water to a concentration of 7 mM, and potassium persulfate was dissolved in water to a concentration of 2.45 mM. The stock solutions were mixed in a 1:1 (v/v) ratio and kept at room temperature for 12–16 h in the dark to prepare the ABTS reaction solution. A volume of 2.8 mL was diluted to 65 mL in acetate buffer at pH 4.5 to obtain the ABTS working solution. The absorbance was measured at 734 nm. Trolox was used as the standard and distilled water as the blank control. The results are expressed as μmol of Trolox equivalents (TE) per g of dry weight (dw) of the sample. Details are reported in Table S1.

### 2.5 Total phenolic content

The total phenolic content (TPC) was determined using Folin–Ciocalteu reagent as previously described with minor modifications.^23^ Briefly, berry extracts (10 μL + 490 μL H_2_O) were reacted with 1 N Folin–Ciocalteu reagent (250 μL, for 5 min) and then neutralized with 1.2 N sodium carbonate (1.25 mL). After 30 min, the absorbance of the resulting solution was measured at 755 nm. Gallic acid was used as the standard. The TPC is expressed as mg of gallic acid equivalents (GAE) per g dw of the sample. Details are reported in Table S2.

### 2.6 Total anthocyanin content

The total anthocyanin content (TAC) was determined by the previously reported pH differential method.^24^ Briefly, two different dilutions were prepared by diluting 1 mL of each extract to 10 mL: one at pH 1 with potassium chloride buffer and the other at pH 4.5 with sodium acetate buffer. The absorbance of the samples was measured at 510 and 700 nm (to correct for haze) against a blank sample consisting of ultra‐pure water. The TAC is expressed as mg of cyanidin 3‐glucoside equivalents per g of sample. Therefore, the maximum absorbance of cyanidin 3‐glucoside (510 nm) and its molar absorptivity (ε = 26,900) were employed. Details are reported in Table S3.

### 2.7 UHPLC‐HRMS analysis

Phenolic compound chromatographic separation and MS analysis were carried out using a previously reported platform based on RP separation using a Kinetex core‐shell C_18_ column (100 mm × 2.1 mm i.d.) and MS analysis with a TOP 5 data‐dependent acquisition (DDA) mode for both low‐ and high‐molecular‐weight phenolic compounds. Details are provided in the Supporting Information. All samples were run in triplicate.

### 2.8 Phenolic compound identification

Raw data obtained from three consecutive injections and the blank sample were processed by Compound Discoverer 3.1 (Thermo Fisher Scientific) using a customized method specifically dedicated to phenolic compound analysis.^25^ Details are provided in the Supporting Information. The identification data for the tentatively identified compounds are discussed in the following sections and summarized in Tables S4–S6 with the related confidence level according to Schymanski *et al*.^26^ Whenever the annotated compounds could be compared to available standards in terms of retention time, accurate mass, and MS/MS spectrum, the compounds were considered identified (confidence level 1). Otherwise, the compounds are tentatively identified (confidence level 2–3).

### 2.9 Identification of phenolic compound degradation products

To gain knowledge on the degradation processes of phenolic compounds after the fermentation process, a customized data processing workflow was set up in Compound Discoverer based on the *Expected Compounds* tool. The most abundant identified aglycones (quercetin, kaempferol, isorhamnetin, myricetin, cyanidin, delphinidin, caffeic acid, ferulic acid, coumaric acid, dihydroxybenzoic acid, and gallic acid) were selected, and their structures were implemented in the *Generate Expected Compounds* tool. Some transformations were chosen among the default ones, e.g., oxidation and desaturation, while others, such as quinone and quinone methide formation, were manually implemented. A maximum of three modifications was considered, and the *Dealkylation* and *Dearylation* tools were not enabled. After spectrum selection and alignment from the raw data files, expected compounds were searched with a mass tolerance of 5 ppm and a minimum peak intensity of 20,000. Detected and expected compounds were then merged since some of the transformation products could have already been reported in the customized database used for phenol identification. The FISh scoring tool, which provides a score for the detected expected compounds and annotates related MS^2^ scans by fragment ion search, was also enabled.

## 3 RESULTS AND DISCUSSION

### 3.1 Phenolic compound composition of *V. Floribundum* berries

Phenolic compounds are a structurally diverse class of compounds, encompassing a wide range of molecular weights and physicochemical properties. Because of the wide range of bond energies in phenolic compound structures (from weak acetal to strong conjugated and aromatic bonds), the acquisition was performed with a three‐step normalized collision energy of 20–50–80 and 20–40–60 for positive and negative ion mode, respectively. Distinct chromatographic runs for each polarity were preferred to polarity switching mode for obtaining a larger number of data points per peak. Final spectrum acquisition and manual validation allowed the identification and tentative identification of 15 and 294 compounds in the native Andean blueberry extract, respectively, including anthocyanins, flavonols, hydroxybenzoic acids, hydroxycinnamic acids, and PACs.

#### 3.1.1 Flavonoid and anthocyanin composition

Manual validation of MS^2^ spectra allowed the tentative identification of 119 compounds belonging to the flavonoid class. The identification of flavonoid aglycones has been largely addressed in HRMS, and, in proper conditions,^25^ several diagnostic product ions can be used for distinguishing several species. Among the several flavonoid subclasses, anthocyanin and flavonols were the most abundant, in terms of both the number of identifications and peak areas (Table S4). In total, 31 anthocyanins were tentatively identified in *V. floribundum* berries, a much larger number compared to previous studies,^15^, ^17^, ^18^ but still lower than reported for *V. myrtillus*.^11^ Cyanidin derivatives were by far the most abundant compounds (70.7%), followed by pelargonidin (14.9%) and delphinidin (12.1%) derivatives (Figure S1A). Minor identified constituents were peonidin and petunidin glycoconjugates (1.7 and 0.7%, respectively). The determined anthocyanin composition was in good agreement with previous studies, which reported cyanidin and delphinidin (but not pelargonidin) as the most abundant aglycones. Vasco *et al*.^15^ identified 7 anthocyanin derivatives, encompassing only cyanidin and delphinidin derivatives. More recently, Esquivel‐Alvarado *et al*.^17^ reported 5 anthocyanin derivatives, still limited to cyanidin and delphinidin glycoconjugates, with similar proportions compared to our results. The results obtained by Esquivel‐Alvarado *et al*. are particularly interesting, as their study was performed by a targeted approach using several analytical standards, including malvidin glycoconjugates, which were determined in other species of the genus *Vaccinium*, but not in *V. floribundum*. This peculiar anthocyanin composition could be effectively used to differentiate *V. floribundum* from other *Vaccinium* species, such as the widespread *V. myrtillus* and *V. corymbosum*. The absence of malvidin derivatives also affects the color of the Andean blueberry extract, which is significantly more reddish (and less purplish) than those of blueberry and bilberry. On the other hand, pelargonidin derivatives have been scarcely reported in blueberries and other berries of the genus *Vaccinium*.^11^, ^27^

Flavonols are the other main flavonoid subclass in the *V. floribundum* extract, with 67 tentatively identified compounds. Among the several aglycones belonging to this class, quercetin derivatives were by far the most abundant with more than 97% of the total flavonol peak area, followed by minor amounts of kaempferol, myricetin, isorhamnetin, and laricitrin derivatives (Figure S1B). The flavonol composition of Andean blueberry is noticeably similar to that of other *Vaccinium* species, except syringetin derivatives, which were not identified in the *V. floribundum* extract.^11^, ^28^ Similar to malvidin, syringetin is a highly *O*‐methylated compound. Their simultaneous absence could indicate a lower degree of methylation in the flavonoid constituents of Andean blueberries compared to European and North American blueberries. These trends are consistent with the results obtained for *Disterigma alaternoides*, another blueberry from the Neotropical realm.^29^

The determination of the position of the glycoconjugation on the flavonol structure is a great analytical challenge when analytical standards are not available. The sugar–aglycone bond can undergo both heterolytic and homolytic cleavage in negative ion mode, producing an aglycone ion [Y_0_]^−^ and a radical aglycone ion [Y_0_‐H]^−^, respectively. Differently from the hydroxyl position on the aromatic rings (e.g., position 7 on the A‐ring or position 4′ on the B‐ring), when a sugar is bound to position 3 (on the non‐aromatic C‐ring of the flavonol structure), the homolytic cleavage is favored.^30^ Based on these shreds of evidence, whenever the radical aglycone ion had a higher abundance than the aglycone ion, the compounds were described as 3‐*O*‐monosaccharide derivatives. Otherwise, the position was not indicated, as positions 7 and 4′ are not distinguishable in HRMS. In agreement with previous findings on other *Vaccinium* species, the majority of flavonols were 3‐*O*‐glycosylated.^11^

#### 3.1.2 Phenolic acid composition

Despite their significant structural variability and interesting biological activities, phenolic acids in *V. floribundum* have been generally neglected to date, even though they constitute the most abundant class of phenolic compounds in *Vaccinium* species.^31^ No more than 7 phenolic acids have been reported so far by previous liquid chromatography (LC)–MS analyses on the phenolic composition of Andean blueberry, encompassing two isomers of chlorogenic acid, one or two caffeoyl shikimic acid isomers, and a few other unknown caffeic acid derivatives.^15^, ^16^, ^18^ Those results, which appear inconsistent with the reported phenolic acid content in blueberries, can be partly explained by the frequent use of MS in positive polarity for the phenolic analysis of anthocyanin‐rich matrices. This strongly acidic class of compound is, in fact, ionized with poor efficiency in positive ion mode. In the present paper, a total of 145 phenolic acids and phenolic acid derivatives have been tentatively identified in the Andean blueberry extract by HRMS analysis in negative ion mode. This number was significantly larger than previously reported for *V. floribundum* and compared to studies on other *Vaccinium* species.^11^, ^28^, ^32^ Detailed data on the tentatively identified compounds are reported in Table S5. Caffeoyl arbutin, chlorogenic acid, caffeoyl acetyl arbutin, caffeoyl shikimic acid, and coumaroyl arbutin presented the highest peak areas. It is worth noting that both caffeoyl arbutin and chlorogenic acid have higher peak areas than any other member of the flavonoid class, in agreement with the reported prevalence of phenolic acids in *Vaccinium* species. Moreover, three of the five most abundant compounds were reported for the first time in *V. floribundum*, as well as any other of the arbutin conjugates. In general, the identified phenolic acid conjugates were mainly hydrophobic hydroxycinnamic derivatives (caffeoyl, coumaroyl, and, to a lesser extent, feruloyl and sinapoyl conjugates) rather than hydrophile hydroxybenzoic derivatives. Arbutin derivatives and quinoyl/shikimoyl conjugates were the most abundant classes of compounds, with about 47% and 39% of the total phenolic acid area, respectively (Figure S2). The most represented subclass of phenolic acids in terms of the number of identifications were instead glycoconjugates of hydroxycinnamic acids, either alone or in combination, with 61 tentatively identified compounds. Gallic acid and its polymeric derivatives (gallotannins and ellagitannins) were scarcely represented, while several minor glycoconjugates of benzoic, hydroxybenzoic, and dihydroxybenzoic acid have been tentatively identified. Despite a large number of identified compounds (79 compounds), phenolic acid glycoconjugates represented just above 8% of the total peak area, possibly due to a large number of minor positional isomers. Among the other minor constituents, three coumaroyl iridoids (compounds 245–247) were tentatively identified; these compounds, which are characteristic of cranberry (*Vaccinium macrocarpon*), are of great interest for their possible role in healing urinary tract infections.^33^

#### 3.1.3 Proanthocyanidin composition

PACs are non‐hydrolyzable polymers of flavanols, mainly (epi)catechin and (epi)gallocatechin, and are distinguished into two subclasses according to their linkage. In particular, A‐type PACs present a double linkage of position 7 and 8 on ring A of the terminal unit to position 2 and 4 on ring C of the extension unit (2β → O → 7; 4β → 8), whereas B‐type PACs present a single interflavanoid bond (4β → 8). Despite being efficiently ionized in both positive and negative ion mode, PACs have been only analyzed in negative polarity for the higher clarity of the MS^2^ spectra and the minor interference of contaminants and noise. In Table S6, detailed data on the 45 tentatively identified PACs are reported. For simplicity, species presenting at least one A‐type interflavanoid bond are commonly defined as A‐type PACs.^34^ Nevertheless, it is worth specifying that, while dimers are clearly distinguished (as there is either one A‐type bond or one B‐type bond), in the case of the other oligomers, there are more than two possibilities, i.e., in the case of trimers, two B‐type bonds, two A‐type bonds, and one for each kind, with the latter two both defined as A‐type PACs. Compared to other PAC‐rich matrices, such as tea,^35^ strawberry,^21^ and even bilberry,^11^ the identified compounds are mostly A‐ and B‐type procyanidins, meaning that they derived from only catechin and epicatechin. A‐type procyanidins were more abundant than B‐type ones in terms of the number of identifications (29 vs. 18) and the total peak area (73% vs. 27%), in good agreement with previous findings for bilberry. Oligomers in the range of 2–6 were tentatively identified, with A‐type trimers and B‐type dimers as the main species.

### 3.2 Transformation of the phenolic molecular species after fermentation

The study on the changes in phenolic compounds following fermentation processes is essential to improve the knowledge on a commonly used practice for wild berry consumption^20^ since fermentation processes are believed to enhance the bioavailability of phenolic compounds by converting polymeric polyphenol to simpler compounds.^36^ It has been demonstrated that lactic acid fermentation of fruits and vegetables produces bioactive compounds with antibacterial, antiinflammatory, antioxidant, and antiviral properties.^37^ However, while several studies have evaluated the biological activities as well as the total phenolic, flavonoid, and anthocyanin content of berries subjected to fermentation processes,^38^ the issue of determining the dynamic changes in phenolic molecular species has been dealt with rarely. Recently, Wu *et al*.^39^ have evaluated the concentration of some selected anthocyanins, phenolic acids, and organic acids in blueberry and blackberry during fermentation over time. Our untargeted approach, which allowed the tentative identification of more than 300 phenolic compounds, was applied to native and fermented samples. Therefore, it was possible to evaluate the dynamic changes of the identified compounds by ratios of the single peak areas between the two sets of samples. Thanks to the *Fill gaps* tool present in Compound Discoverer, compounds present with low abundances in one of the two sets of samples (and higher abundance in the other set) could still be identified. Whenever the peaks were absent in one of the two sets of samples, the noise level was chosen as the area, making it still possible to evaluate the ratios (none of the two peak area values were therefore zero). The ratios are reported in Tables S4–S6 for all tentatively identified compounds. In Figure S3, two exemplary chromatograms of the native and fermented berry extracts are shown.

For a more comprehensive evaluation of the results, the antioxidant activity, TPC, and TAC of both native and fermented berries were measured. These are reported in Table 1.

**TABLE 1:** Total phenolic content (TPC), total anthocyanin content (TAC), and antioxidant activity (ABTS), measured for native and fermented *V. floribundum* Kunth berry

	TPC (mg gallic acid/g dw)	TAC (mg cyanidin 3‐glucoside/g dw)	ABTS (μmol TE/g dw)
Native berry	45 ± 3	14.5 ± 1.9	276 ± 2
Fermented berry	38 ± 3	1.0 ± 0.5	288 ± 2

**FIGURE 1:**
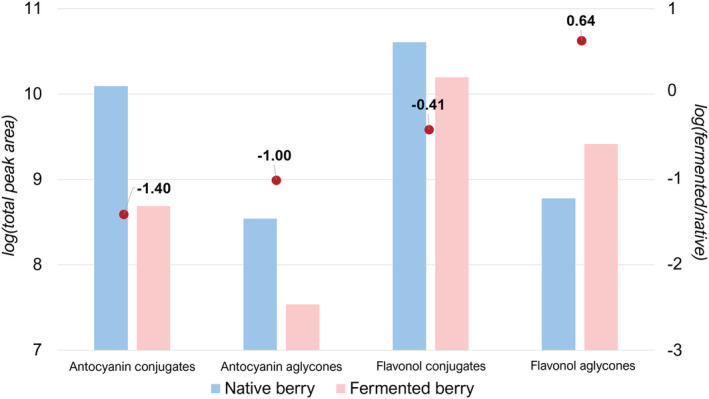
Bar chart representing the total peak areas in the logarithmic scale of anthocyanin and flavonol conjugates and aglycones tentatively identified in native and fermented berry and the logarithm of the ratio fermented/native for each class

The TPC and TAC of the native berry are in line with previously obtained results for hydro‐organic phenol extracts of other *Vaccinium* species.^40^, ^41^ However, the results reported in the literature are often significantly discordant. Therefore, the results are discussed by comparing the native and fermented berries rather than in relation to previous studies. Indeed, the fermentation process enhances the antioxidant power while lowering the polyphenol content due to hydrolysis and oxidation reactions.^42^ Anthocyanins are mainly degraded after the fermentation process, resulting in the brownish color of the fermented berry compared to the bright red color of the native one. In Figure 1, the total peak areas of the two main flavonoid classes are reported for native and fermented berry, along with their ratios on a logarithmic scale.

Confirming the results obtained by the spectrophotometric assay, anthocyanins were extensively degraded during fermentation, with ratios of −1.4 and −1 for conjugates and aglycones in logarithmic scale, which correspond to a 25‐ and 10‐fold decrease, respectively. Conversely, flavonol conjugates showed a much lower ratio of −0.41 (which corresponds to a 2.5‐fold decrease), meaning that flavonol derivatives are much more preserved and less sensitive to heat and oxidative phenomena. Free flavonols presented a positive value of the logarithmic ratio, meaning that their concentrations were higher after the fermentation process (about a 4‐fold increase), possibly due to hydrolysis reactions occurring on the glycosyl acetal bonds. Whereas the flavonol aglycones appear relatively stable under fermentation, free anthocyanins were extensively degraded. Anthocyanin thermal degradation is based on cleavage of the C‐ring of the anthocyanin, generating a phenolic acid in correspondence to the B‐ring (which varies according to the single compound) and phloroglucinaldehyde in correspondence to the A‐ring (which could rapidly oxidize to phloroglucinol carboxylic acid) as shown in Figure S4.^43^ The extremely high increases of some phenolic acids were believed to be mostly the result of these degradation reactions rather than deriving from hydrolysis of phenolic acid conjugates. Phloroglucinol carboxylic acid, which was produced by all anthocyanins, showed a 26‐fold increase, protocatechuic acid (which is derived from cyanidin) presented a 19‐fold increase, gallic acid (deriving from delphinidin) showed a 4‐fold increase, methyl‐protocatechuic acid (deriving from peonidin) showed a 7‐fold increase, and methyl‐gallic acid (deriving from petunidin) showed a 13‐fold increase. Similar to flavonoids, phenolic acid conjugates were extensively hydrolyzed, with a subsequent increase of free hydroxycinnamic acids comparable to that of free flavonols.

In Table 2, the ratios of the peak areas of PACs between fermented and native samples are shown as percentage values. At first glance, there seems to be a correlation between the stability and the length of the PACs, with higher percentage values for shorter oligomers (from 15.9% of dimers to 5.2% of hexamers). However, by separating the contribution of A‐ and B‐type PACs, the results were completely different. B‐type PACs were equally degraded during fermentation regardless of the oligomers' length. Conversely, A‐type PAC degradation was directly proportional to the length of the oligomers. As mentioned in the previous sections, A‐type PACs with three or more units generally present both the double A‐type interflavanoid bond and the single B‐type interflavanoid bond. Therefore, the longer the A‐type oligomer is, the more it is similar to a B‐type PAC, with the result that A‐type pentamers and hexamers have the same percentage values as B‐type ones. Not unexpectedly, the double A‐type interflavanoid bonds render the oligomers more stable, with A‐type dimers (which present a single A‐type bond) being the most stables of the series.

**TABLE 2:** Ratios of the total peak areas of A‐ and B‐type proanthocyanidins (PACs) in fermented and native berry samples expressed as percentage values

	PACs	A‐type PACs	B‐type PACs
Dimers	15.9 ± 6.6%	21.4 ± 6.7%	5.4 ± 2.7%
Trimers	13.7 ± 6.9%	14.8 ± 7.8%	5.6 ± 0.8%
Tetramers	6.5 ± 3.6%	7.1 ± 3.8%	5.4 ± 2.5%
Pentamers	5.4 ± 2.7%	5.6 ± 3.1%	5.1 ± 2.0%
Hexamers	5.2 ± 1.7%	5.0 ± 1.7%	5.7 ± 2.1%
Total	12.4 ± 4.8%	15.0 ± 5.8%	5.4 ± 2.2%

### 3.3 Identification of polyphenol oxidation products

Whereas anthocyanin degradation pathways usually generate phenolic acids, which are already included in the database employed for the suspect screening data analysis, flavonols and hydroxycinnamic acids are typically oxidized to quinones.^44^ Quinones are commonly formed by desaturation of *o‐* and *p‐*dihydroxybenzene rings, generating *o‐* and *p‐*quinones, respectively. Oxidation reactions could occur before desaturation when *o‐* and *p‐*dihydroxybenzene rings are not present in the structure of the molecule. For this purpose, a dedicated data processing workflow was set up in Compound Discoverer by enabling the *Generate expected compounds* tool. Oxidation (+1 oxygen atom) and desaturation reactions (−2 hydrogen atoms) were chosen among the default transformations, while quinone formation (+1 oxygen, −2 hydrogens) was manually implemented.

Moreover, the structures of the most common aglycones were implemented to the method (kaempferol, quercetin, myricetin, isorhamnetin, cyanidin, coumaric acid, caffeic acid, ferulic acid, sinapic acid, hydroxybenzoic acid, dihydroxybenzoic acid, and gallic acid). Raw data files were re‐processed, and the extracted expected compounds were manually identified. In Table 3 and Table S7, the 13 annotated expected compounds are reported.

**TABLE 3:** Annotated phenolic compound quinones in fermented *Vaccinium floribundum* Kunth berry extract

Name	Retention time	Formula	Molecular weight	Transformation	Parent compound
Hydroxycaffeoquinone	3.08	C_9_H_6_O_5_	194.0215	Quinone formation	Caffeic acid
Caffeoquinone	3.42	C_9_H_6_O_4_	178.0266	Desaturation	Caffeic acid
				Quinone formation	Coumaric acid
Ferulic acid quinone	4.89	C_10_H_8_O_5_	208.0372	Quinone formation	Ferulic acid
				Desaturation	Hydroxyferulic acid
Ferulic acid quinone	6.16	C_10_H_8_O_5_	208.0372	Quinone formation	Ferulic acid
				Desaturation	Hydroxyferulic acid
Cyanidin quinone	6.22	C_15_H_9_O_7_^+^	301.0343	Desaturation	Cyanidin
Myricetin *o*‐quinone	12.83	C_15_H_8_O_8_	316.0219	Desaturation	Myricetin
Quercetin *p*‐quinone methide	17.34	C_15_H_8_O_7_	300.0270	Desaturation	Quercetin
				Quinone formation	Kaempferol
Quercetin *o*‐quinone	18.68	C_15_H_8_O_7_	300.0270	Desaturation	Quercetin
				Quinone formation	Kaempferol
Isorhamnetin *p*‐quinone methide	19.67	C_16_H_10_O_7_	314.0427	Desaturation	Isorhamnetin
Kaempferol *p*‐quinone methide	20.24	C_15_H_8_O_6_	284.0321	Desaturation	Kaempferol
Quercetin *p*‐quinone methide isomer	20.33	C_15_H_8_O_7_	300.0270	Desaturation	Quercetin
				Quinone formation	Kaempferol
Quercetin *p*‐quinone methide isomer	20.55	C_15_H_8_O_7_	300.0270	Desaturation	Quercetin
				Quinone formation	Kaempferol
Quercetin *p*‐quinone methide isomer	21.96	C_15_H_8_O_7_	300.0270	Desaturation	Quercetin
				Quinone formation	Kaempferol

**FIGURE 2:**
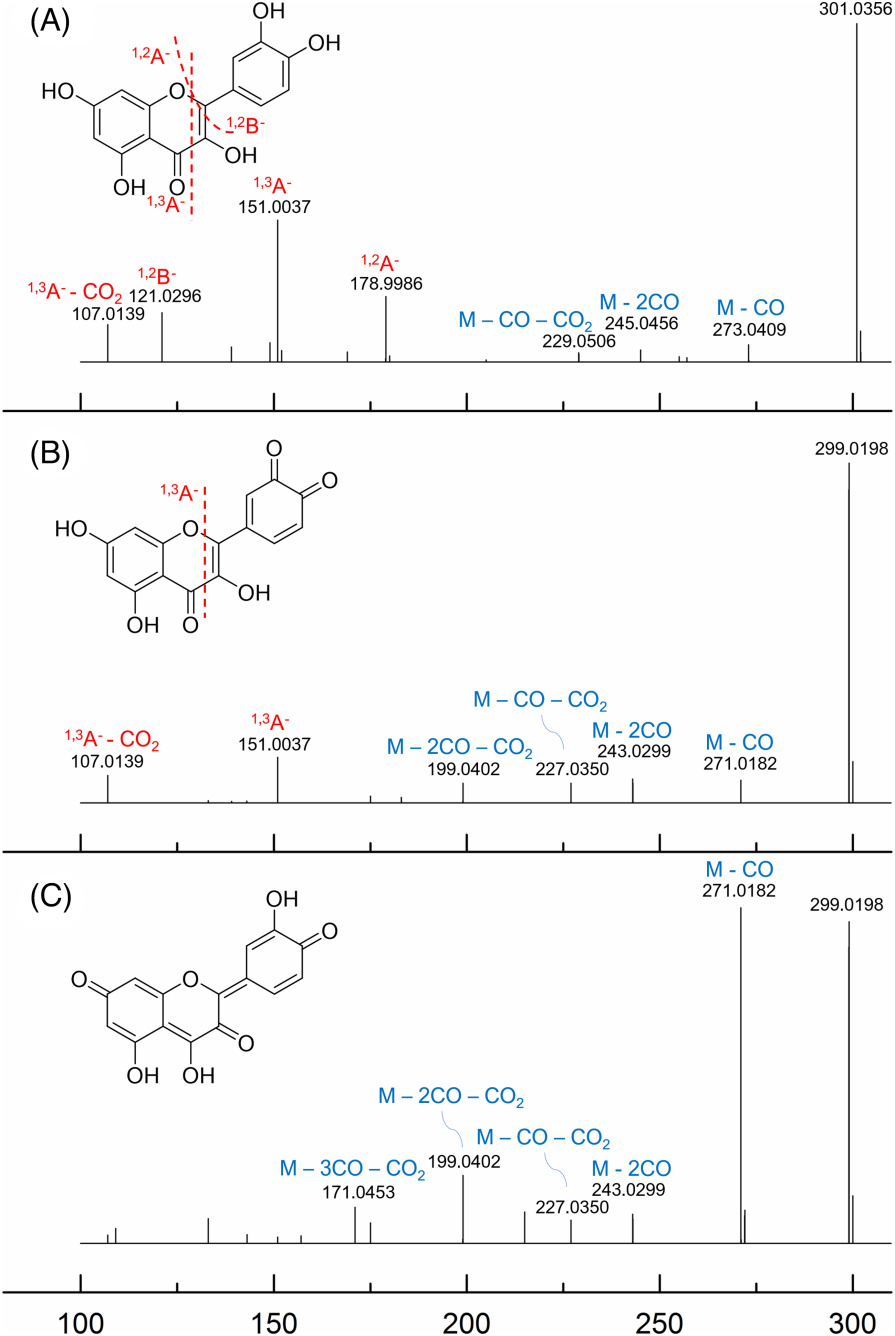
MS^2^ spectrum of quercetin analytical standard (A) and two of the tentatively identified quinone derivatives, quercetin *o*‐quinone (B) and quercetin *p*‐quinone methide (C)

Four phenolic acid quinones were tentatively identified, the most abundant being caffeoquinone. Caffeoquinone could be derived by desaturation of caffeic acid and oxidation followed by desaturation of coumaric acid (the two main phenolic acids in *V. floribundum* extract), while hydroxycaffeoquinone could only derive from caffeic acid by prior oxidation. Two different isomers of ferulic acid quinone were reported, likely deriving from ferulic and hydroxyferulic acid. Unsurprisingly, quinone derivatives of sinapic acid were not reported, as there are no sites on the structure of sinapic acid prone to quinone formation (two hydroxyl groups in either *ortho* or *para*). In the case of flavonoids, *o‐*quinones could be generated on C‐rings, while *p‐*quinone methides are generated alongside the conjugated structure of the flavone. In Figure 2, MS^2^ spectra of quercetin, quercetin *o*‐quinone, and quercetin *p*‐quinone methide are reported. Quercetin undergoes minor neutral losses of CO and CO_2_ (*m*/*z* 273.0409, 245.0456, and 229.0506) and more significant retro Diels–Alder (RDA) fragmentation pathways (*m*/*z* 178.9986, 151.0037, 121.0296, and 107.0139). Quercetin *o*‐quinone was tentatively identified thanks to RDA product ions, which demonstrate that the A‐ring section of the molecule has not changed following the oxidation (Figure 2B). In contrast, quercetin *p*‐quinone methide, which has four isomers, does not show RDA product ions (Figure 2C). For both quinones, however, neutral losses were more significant than in the case of quercetin, likely due to the presence of several ketone groups alongside the structures. Myricetin *o*‐quinone and *p*‐quinone methides of kaempferol and isorhamnetin were tentatively identified with the same logic. Quercetin and myricetin present, in fact, two hydroxyl group in ortho on the C‐ring, which could undergo desaturation and generate *o*‐quinones. Conversely, kaempferol and isorhamnetin cannot generate *o*‐quinones if prior oxidation of the C‐ring does not occur. Quercetin quinones could effectively be derived by preliminary oxidation of kaempferol. However, as similar oxidation products of isorhamnetin were not reported, it is unlikely that kaempferol undergoes C‐ring oxidation rather than desaturation to generate the *p*‐quinone methide.

Overall, *V. floribundum* exhibited a polyphenol composition that is generally similar to other species of blueberries, with high concentrations of anthocyanins, flavonols, and hydroxycinnamic acids. Still, *V. floribundum* showed several peculiarities, e.g., the absence of malvidin conjugates and numerous pelargonidin derivatives, indicating that the extreme growing conditions have a significant effect on the composition of phenolic compounds. For the first time, the transformations of phenolic compounds after lactic fermentation were investigated. Despite a significantly lower TPC and the almost complete degradation of anthocyanins, the fermented berry exhibited a higher antioxidant activity compared to the fresh berry. Glycoconjugations were mostly hydrolyzed following fermentation, and higher concentrations of free flavonoids and phenolic acids were present. Further studies are required to evaluate the fresh and fermented berry phenolic extracts in vivo, as the bioavailability of phenolic compounds is strictly correlated to their dimension and degree of glycosylation.

## Supporting information


**Figure S1.** Pie chart representing (A) the anthocyanin and (B) the flavonol composition of *Vaccinium floribundum* Kunth extract. The percentages are calculated based on the total peak areas.
**Figure S2.** Pie chart representing the phenolic acid composition of *Vaccinium floribundum* Kunth extract. The percentages are calculated based on the total peak areas.
**Figure S3.** Exemplary chromatograms of (A) native and (B) fermented *Vaccinium floribundum* berry extracts recorded in positive ion mode. The degradation of the anthocyanins in the retention time interval 3–7 min is evident.
**Figure S4.** Degradation pathways of cyanidin and delphinidin.

**Table S1.** ABTS antioxidant assay
**Table S2.** Total phenol content (Folin–Ciocalteu method)
**Table S3.** Total anthocyanin content
**Table S4.** Detailed data on the tentatively identified flavonoids and anthocyanins
**Table S5.** Detailed data on the tentatively identified phenolic acids
**Table S6.** Detailed data on the tentatively identified proanthocyanidins
**Table S7.** Detailed data on the tentatively identified expected compounds

## Data Availability

The data that supports the findings of this study are available in the supplementary material of this article. Further data are available from the corresponding author upon reasonable request.
