# Supplementary material for: Detailed investigation of the composition and transformations of phenolic compounds in fresh and fermented Vaccinium floribundum berry extracts by high‐resolution mass spectrometry and bioinformatics
Source: Phytochem Anal. 2022 Jan 21;33(4):507–16. doi: 10.1002/pca.3105 (PMC9543071; doi:10.1002/pca.3105)
Supplement: Supplementary file 1 — Figure S1. Pie chart representing (A) the anthocyanin and (B) the flavonol composition of Vaccinium floribundum Kunth extract. The percentages are calculated based on the total peak areas. Figure S2. Pie chart representing the phenolic acid composition of Vaccinium floribundum Kunth extract. The percentages are calculated based on the total peak areas. Figure S3. Exemplary chromatograms of (A) native and (B) fermented Vaccinium floribundum berry extracts recorded in positive ion mode. The degradation of the anthocyanins in the retention time interval 3–7 min is evident. Figure S4. Degradation pathways of cyanidin and delphinidin. [file PCA-33-507-s002.docx]

**Supporting Information**

**Detailed investigation of the composition and transformations of phenolic compounds in fresh and fermented Vaccinium Floribundum berry extracts by high-resolution mass spectrometry and bioinformatics**

Andrea Cerrato^1,1^, Susy Piovesana^1,1^, Sara Elsa Aita^1^, Chiara Cavaliere^1,*^, Simona Felletti^2^, Aldo Laganà^1,3^, Carmela Maria Montone^1^, Celia Vargas-de-la-Cruz^4^, Anna Laura Capriotti^1^

^1^ Department of Chemistry, Sapienza University of Rome, Piazzale Aldo Moro 5, 00185 Rome, Italy.

^2^ Department of Chemistry and Pharmaceutical Sciences, University of Ferrara, via L. Borsari 46, 44121 Ferrara, Italy.

^3^ CNR NANOTEC, Campus Ecotekne, University of Salento, Via Monteroni, 73100 Lecce, Italy

^4^ Faculty of Pharmacy and Biochemistry, Academic Department Pharmacology, Toxicology and Bromatology, Centro Latinoamericano de Enseñanza e Investigación en Bacteriología Alimentaria-CLEIBA, Universidad Nacional Mayor de San Marcos, Lima-Perú.

^1^ These authors contributed equally to the paper

***Corresponding author**

Dipartimento di Chimica

Sapienza Università di Roma

Piazzale Aldo Moro 5, 00185 Rome, Italy

E-mail: [chiara.cavaliere@uniroma1.it](mailto:chiara.cavaliere@uniroma1.it)

Phone : +39 06 4991 3834

**UHPLC-HRMS analysis**

Phenolic compound chromatographic separation carried out by a Vanquish binary pump H (Thermo Fisher Scientific, Bremen, Germany), equipped with a thermostated autosampler and column compartment, on a Kinetex core-shell C_18_ column (100 mm × 2.1 mm i.d.) with a particle size of 2.6 µm (Phenomenex, Torrance, CA, USA) at 40 °C and at a flow-rate of 600 µL min^-1^. The injection volume was 10 µL. The mobile phases consisted of H_2_O/HCOOH (99.9:0.1, *v*/*v*; phase A) and ACN/HCOOH (99.9:0.1, *v/v*; phase B). The elution gradient was optimized in a previous study.^21^ The chromatographic system was coupled to a Q Exactive hybrid quadrupole-Orbitrap mass spectrometer (Thermo Fisher Scientific) with a heated ESI source.

The detection was conducted in TOP 5 data-dependent acquisition (DDA) mode for both low- and high-molecular-weight phenolic compounds. An exclusion list containing the most intense ions detected in a blank sample consisting of H_2_O/MeOH (90:10, *v*/*v*) was added to the mass-spectrometric method. For low-molecular-weight phenolic compound analysis (flavonoids, anthocyanins, and phenolic acids) and high-molecular-weight polyphenol analysis (tannins), MS data were acquired in the range 150-1000 *m/z* and 300-2000 *m/z*, respectively, with a resolution (full width at half maximum, FWHM, at *m/z* 200) of 70,000. In full scan mode, the automatic gain control (AGC) target value was 200,000, and the maximum ion injection time was 100 ms. The isolation window width was 2 *m*/*z*. Tandem MS (MS^2^) fragmentation was performed with a resolution (FWHM, at *m/z* 200) of 35,000 with AGC target value set at 100,000 and dynamic exclusion set to 3 s. Stepped collision energy fragmentation was achieved in the higher energy collision dissociation (HCD) cell at three values of normalized collision energy (NCE), namely, 20-50-80 NCE in positive ion mode and 20-40-60 NCE in negative ion mode based on the results of a previous study.^25^

**Phenolic compound identification**

Raw data obtained from three consecutive injections and the blank sample were processed by Compound Discoverer 3.1 (Thermo Fisher Scientific) using a customized method specifically dedicated to phenolic compound analysis.^25^ The software is based on a system of blocks and nodes which can be customized by the user for the development of specific data-processing methods. Customized databases generated by combining free phenolic compounds (aglycones) with a series of sugars, aliphatic and aromatic acids, and complete of IDs, accurate masses, and molecular formulas were implemented in the *mass list* feature for the automatic matching of extracted *m/z* ratios. Moreover, detailed HCD fragmentation spectra for flavonoids and phenolic acids were implemented in the *compound class scoring* section for automatic MS^2^ spectra matching, and parameters for *predict composition* tool were adapted to phenolic compounds. Extracted *m/z* from the raw chromatograms were grouped, aligned, and filtered to remove background compounds found in the blank sample, compounds whose *m/z* were not associated with compounds present in the databases, and those to which no MS^2^ spectra were associated. Filtered compounds were manually validated by matching fragmentation spectra to those of available standards or spectra reported in the literature. When data were lacking, phenolic compounds were tentatively identified according to the characteristic fragmentation spectra.


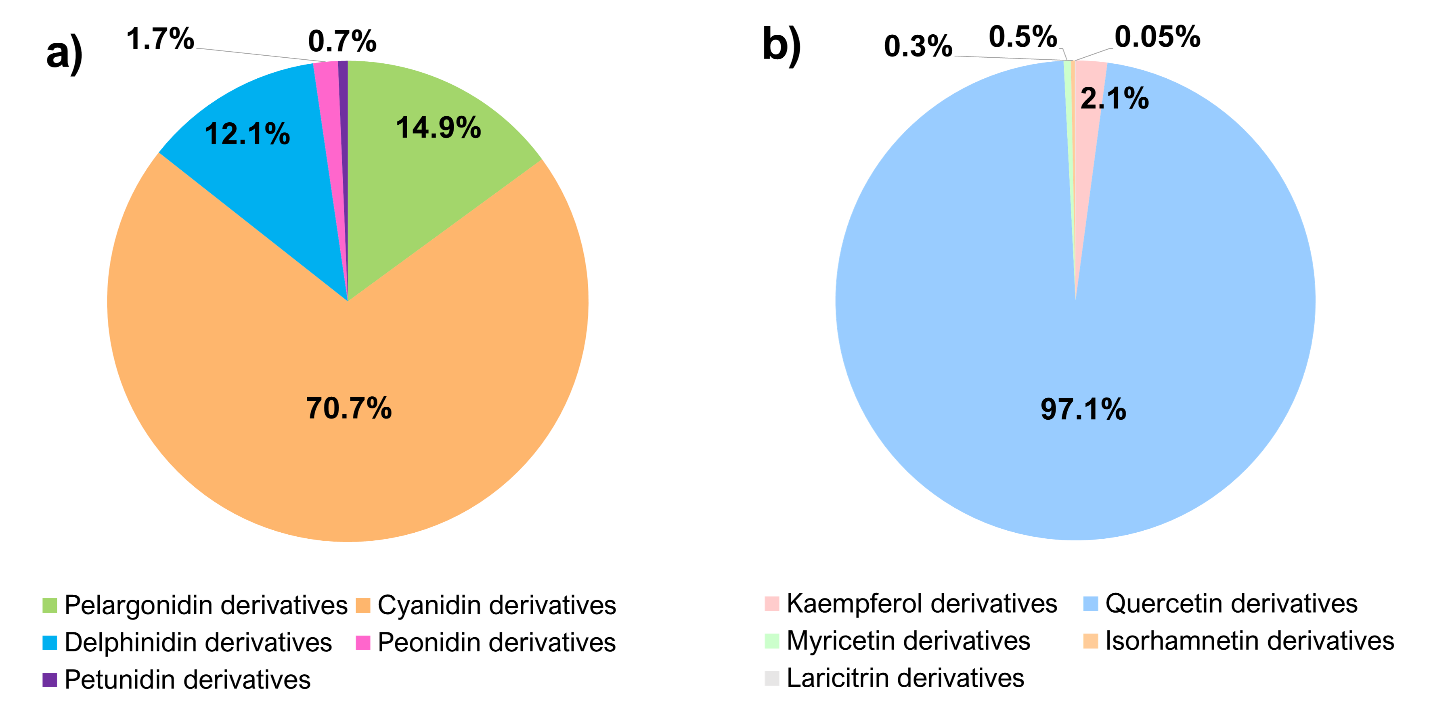


**Figure S1.** Pie chart representing (a) the anthocyanin and (b) the flavonol composition of *Vaccinium floribundum* Kunth extract. The percentages are calculated based on the total peak areas.


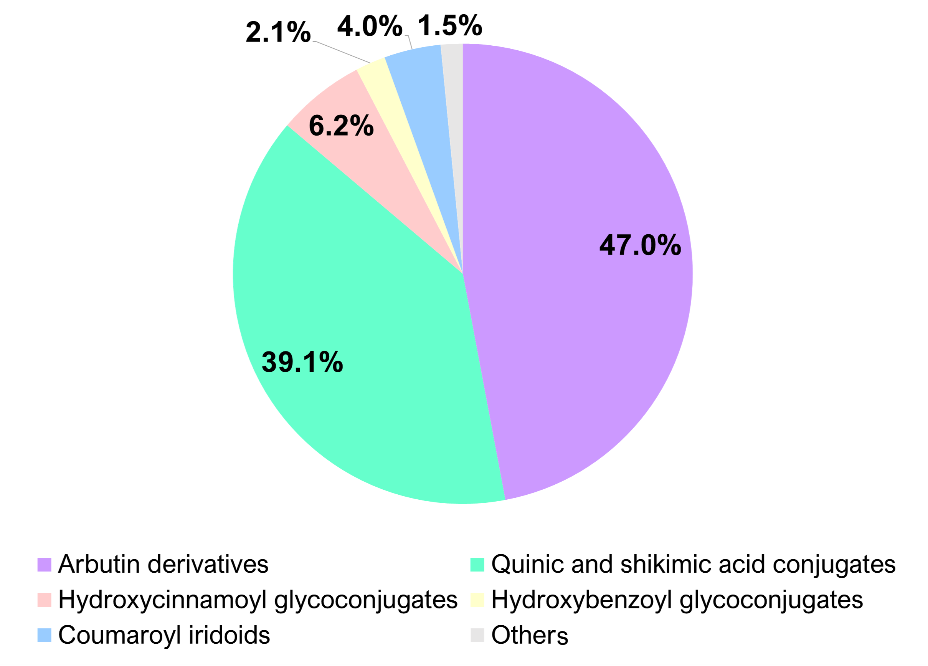


**Figure S2.** Pie chart representing the phenolic acid composition of *Vaccinium floribundum* Kunth extract. The percentages are calculated based on the total peak areas.

**
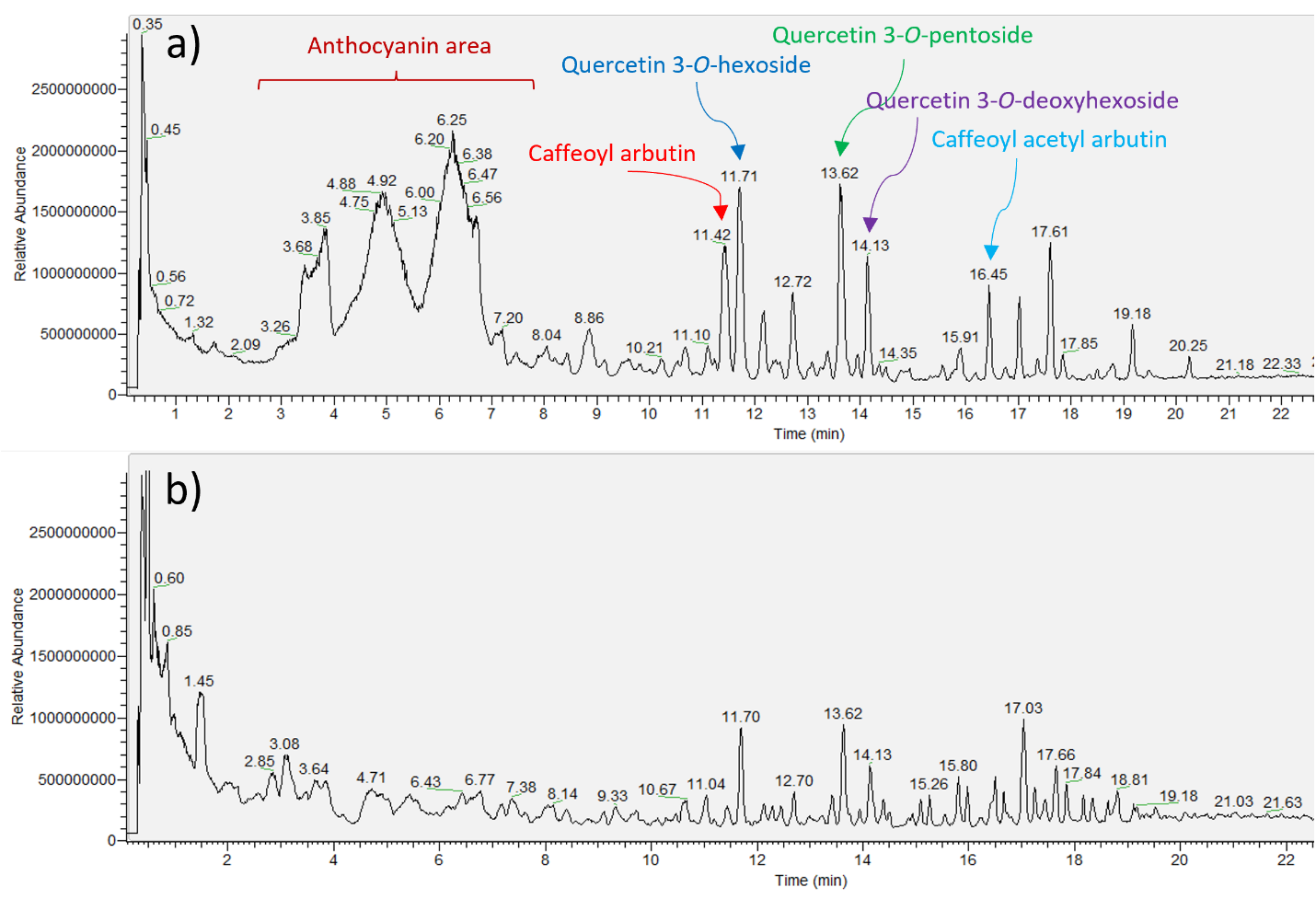
**

**Figure S3.** Exemplary chromatograms of (a) native and (b) fermented V. floribundum berry extracts recorded in positive ion mode. It is evident the degradation of the anthocyanins in the rt interval 3-7 mins.


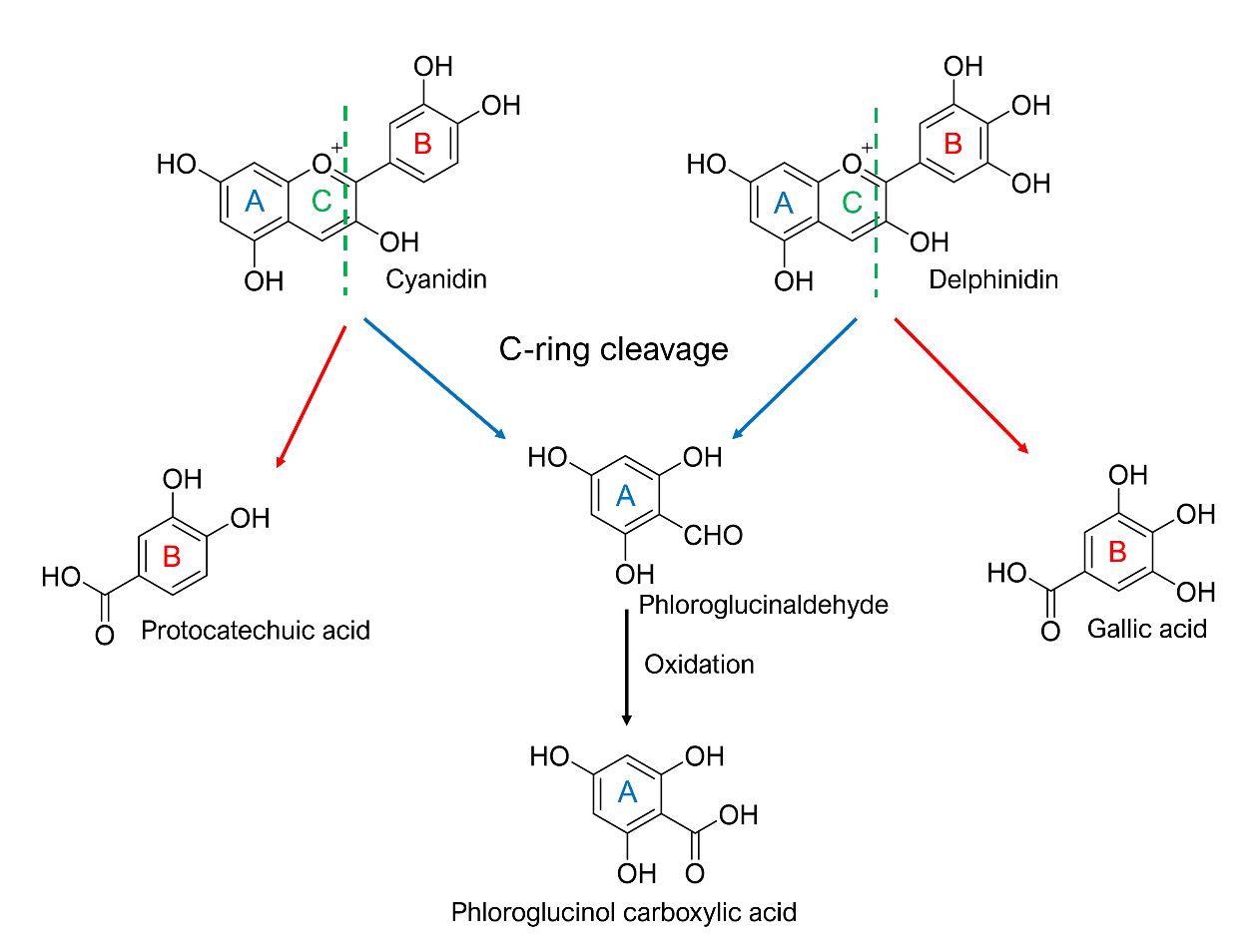


**Figure S4.** Degradation mechanism pathways of cyanidin and delphinidin.
